# AI-Assisted Clinical Documentation in Routine Danish General Practice: Quantitative Pre-Post Study

**DOI:** 10.2196/94431

**Published:** 2026-10-02

**Authors:** Louise Nørgaard Olsen, Philipp Harbig, Anna Bay Laurberg, Jacob Laurberg, Morten Haaning Charles

**Affiliations:** 1 Department of Internal Medicine, Regional Hospital Viborg, Heibergs Allé 5A, Viborg, 8800, Denmark, 45 60211057; 2 Department of Public Health, Research Unit for General Practice, Aarhus University, Aarhus, Central Jutland, Denmark; 3 General Practice, Aarhus, Denmark; 4 Faculty of Health, Aarhus University, Aarhus, Denmark; 5 Steno Diabetes Center Aarhus, Aarhus University Hospital, Aarhus, Central Jutland, Denmark; 6 Department of Clinical Medicine, Aarhus University, Aarhus, Central Jutland, Denmark

**Keywords:** artificial intelligence, AI, clinical documentation, general practice, primary care, ambient documentation, electronic health records, technology acceptance, clinical workflow

## Abstract

**Background:**

Administrative workload in general practice limits time for direct patient care. AI-assisted documentation has been proposed as a way to reduce the documentation burden, but evidence from routine primary care settings remains limited.

**Objective:**

This study aimed to evaluate general practitioners’ (GPs) acceptance of AI-assisted documentation and its association with documentation time and clinical note quality in routine Danish general practice.

**Methods:**

We conducted a quantitative pragmatic pre-post quality improvement evaluation in Danish general practice. A total of 20 GPs documented 239 consultations before and 236 consultations after implementation of an AI-assisted documentation system. Documentation quality, structure, clinical clarity, and documentation time categories were self-assessed using standardized audit forms completed immediately after each consultation. Technology acceptance and usability were assessed using the technology acceptance model (TAM) and the System Usability Scale (SUS).

**Results:**

Self-assessed documentation structure increased from 3.99 to 4.45, while self-reported documentation time categories decreased from 2.85 to 2.29. Technology acceptance and usability were high (TAM domain means 3.76-4.19; SUS mean 77.5). GP-level paired analyses showed moderate improvements in structure and clarity and a reduction in documentation time. Combined blinded external assessments showed higher postimplementation scores for quality, structure, and clinical clarity, although reviewer-specific ratings diverged, and interrater reliability was low. The association between documentation time and perceived quality was negligible. TAM and SUS indicated high clinician acceptance.

**Conclusions:**

AI-assisted documentation was associated with lower self-reported documentation time categories while maintaining or modestly improving perceived clinical note quality. These findings support the feasibility of AI-assisted documentation in primary care, while highlighting the need for controlled studies with objective time measurement and longer follow-up.

## Introduction

Administrative workload in general practice has increasingly constrained the time available for direct patient care and continuity of clinical interaction [1,2]. A substantial proportion of this burden relates to documentation and maintenance of electronic health records (EHRs), which often compete with patient-facing time and contribute to clinician workload and dissatisfaction [3]. In response, digital health innovations aimed at supporting documentation processes have received growing attention.

Recent advances in AI have enabled the development of so-called “ambient” or AI-assisted documentation systems that capture clinician-patient dialogue, generate real-time transcripts, and produce draft clinical notes for clinician review and approval [4-6]. These systems are designed to support clinical documentation by generating draft clinical notes for clinician review and approval rather than functioning as autonomous documentation systems. The clinician retains full responsibility for reviewing, editing, and approving the final clinical note. International studies suggest that AI-assisted documentation may reduce perceived documentation burden and support workflow efficiency without compromising documentation quality [1,4,5,7]. However, the existing evidence base is heterogeneous, and many studies are conducted in selected settings or under experimental conditions.

Implementation of AI-based documentation in general practice raises specific considerations related to trust, usability, data protection, and professional accountability. In European primary care settings, compliance with the General Data Protection Regulation (GDPR), transparency of data handling, and clinician control over documentation are central prerequisites for adoption [8,9]. Qualitative studies indicate that general practitioners (GPs) perceive both opportunities and reservations regarding AI-supported documentation, emphasizing the importance of usability, reliability, and a clear “human-in-the-loop” workflow [9,10].

Despite growing interest, evidence from routine primary care workflows remains limited, particularly from Scandinavian general practice. Moreover, few studies have examined whether clinicians’ self-assessments of AI-assisted documentation quality correspond with independent professional evaluations. External blinded assessment of documentation quality is rarely included in implementation studies, yet such evaluation is relevant to address concerns regarding potential bias in self-reported outcomes.

The aim of the present study was therefore to evaluate the feasibility of implementing an AI-assisted documentation system in routine Danish general practice. Specifically, we aimed to assess (1) GPs’ acceptance and perceived usability of the system; (2) perceived changes in documentation time; and (3) changes in clinical note quality, structure, and clinical clarity. To strengthen the evaluation beyond self-assessment alone, we additionally compared GPs’ ratings of documentation quality with blinded assessments performed by independent physicians. By combining clinician-reported outcomes with external evaluation under real-world conditions, this study aimed to contribute pragmatic evidence on the role of AI-assisted documentation in everyday primary care practice.

## Methods

### Study Design and Setting

We conducted a quantitative pragmatic pre-post quality improvement evaluation of the implementation of an AI-assisted clinical documentation system in routine Danish general practice. The primary objective was to evaluate clinician acceptance, perceived usability, and integration of the system into routine clinical workflows rather than patient-level clinical outcomes. The study was carried out within a regional primary care quality cluster (“klynge” in Danish) comprising 7 GP clinics and 20 participating GPs. The cluster had been an established structure for several years before the study and was not formed specifically for this project. It was based on administrative and geographic criteria and was not established based on interest in digital health or information technology, thereby reflecting a routine primary care setting. Participation was voluntary and offered to all GPs within the quality cluster. The participating practices included both solo and partnership practices, but formal measures of practice characteristics, patient population, and digital maturity were not collected. Data collection was conducted between October 2025 and November 2025.

### Selection of the AI-Assisted Documentation System

Prior to study initiation, several AI-assisted documentation systems available for Danish general practice were informally explored by the project group with regard to usability and fit with routine clinical workflows. This was a pragmatic local assessment rather than a formal comparative evaluation or procurement process. Noteless (Noteless AS) was selected pragmatically by the project group based on perceived suitability for implementation in routine Danish general practice and its availability for use within the participating quality improvement initiative. The selection was not based on a formal comparative evaluation of available AI-assisted documentation systems. Consequently, the findings should be interpreted as specific to this system, version, and implementation context and may not be directly transferable to other AI-assisted documentation systems.

### Participants and Consultations

Participating GPs were instructed to document up to 12 consecutive in-person consultations before implementation of the AI-assisted documentation system. Following system installation, a 2-week familiarization period was allowed to enable integration into routine clinical workflows. After this period, GPs documented up to 12 consecutive in-person consultations using the AI-assisted system.

During both periods, GPs aimed to document 12 consecutive in-person consultations in each period; consultations could be excluded at the GP’s discretion if deemed unsuitable, and inclusion continued until 12 eligible consultations were reached when feasible. Documentation time categories and note quality ratings were completed by the participating GP immediately after each documented consultation. No restrictions were placed on patient characteristics or consultation type within these in-person consultations, and participating GPs were encouraged to use the AI-assisted documentation system whenever they considered it appropriate within routine clinical practice. Due to pragmatic clinical conditions, incomplete registrations, and missing ratings, the number of evaluable notes varied between GPs. Only notes with complete outcome ratings were included. GP-level analyses were therefore based on the mean of available preimplementation and postimplementation notes for each GP. This pragmatic approach was chosen to minimize disruption to routine clinical workflows and reduce the additional documentation burden associated with study participation. Consequently, participating GPs were not required to document reasons for excluding individual consultations.

### Intervention: AI-Assisted Documentation Workflow

The intervention consisted of using the AI-assisted documentation system Noteless during routine in-person consultations. Immediately before each eligible consultation, the GP initiated a secure personal web browser session and activated real-time transcription. The system captured clinician-patient dialogue to generate a draft clinical note at the end of the consultation. The GP then reviewed and edited the AI-generated draft before final approval and storage in the EHR. The AI-assisted documentation system operated as a web-based application and was not directly integrated with the participating practices’ EHR systems. After reviewing and editing the AI-generated draft, the GP manually transferred the final note into the local EHR by copying and pasting. The specific EHR systems used by the participating practices were not recorded.

Audio capture was used solely to enable real-time transcription and draft note generation. Complete audio recordings were not retained. According to the implemented workflow, audio data were deleted immediately, while transcription outputs and generated draft notes were temporarily retained within a European Union (EU)–based cloud environment for up to 24 hours to allow clinician review and editing, after which they were deleted. These transcription outputs and draft notes were not accessible to the vendor after deletion and were not used for AI model training. Evaluation data were not made accessible to the vendor, and clinical notes used for external assessment were deidentified before sharing.

### Data Collection and Outcomes

Data were collected using structured audit forms completed by participating GPs, along with the technology acceptance model (TAM) questionnaire and the System Usability Scale (SUS).

#### Primary Outcomes (Pre-Post)

The following 4 outcomes were assessed both before and after implementation using structured audit forms: overall satisfaction with the quality of the clinical note, whether the clinical note was well structured, whether another health care provider could understand the patient’s clinical course based on the note, and documentation time. The first 3 outcomes were rated on 5-point Likert scales, with higher scores indicating more favorable assessments. Documentation time was recorded using 5 ordinal categories (<1 minute, 1 minute, 2-3 minutes, 4-5 minutes, and >5 minutes) and coded from 1 to 5, with lower values indicating shorter documentation time.

All primary outcomes were self-assessed by participating GPs for each documented consultation.

#### Postimplementation Additional Outcome

After implementation, GPs additionally rated the perceived impact on time available for direct patient care using a 5-point Likert scale. As no corresponding preimplementation measure was available, this outcome was analyzed descriptively only.

### Technology Acceptance and Usability

Technology acceptance was assessed using a modified TAM questionnaire comprising 12 items covering 4 domains: perceived usefulness (4 items), perceived ease of use (4 items), attitude toward use (2 items), and behavioral intention to use (2 items). All TAM items were rated on a 5-point Likert scale ranging from strongly disagree to strongly agree. The TAM questionnaire was translated into Danish by 1 of the investigators for use in this quality improvement project and did not constitute a formally validated Danish version. System usability was assessed using the standard 10-item SUS, also using a 5-point Likert response scale. Participants completed the original English SUS questionnaire, while a Danish translation was provided solely as a reference to support comprehension if needed. The questionnaire explicitly instructed participants to answer the original English items and to consult the Danish translation only if they were uncertain about the meaning of the English wording. SUS responses were converted to a total score ranging from 0 to 100, with higher scores indicating better perceived usability.

### Blinded External Assessment of Clinical Notes

To reduce potential self-assessment bias, all preimplementation and postimplementation clinical notes were deidentified and randomly ordered. Patient-identifying information was manually removed before the notes were assigned unique study ID numbers. The deidentified notes were then randomly ordered and compiled into PDF documents for independent review. The reviewers did not access the Noteless system or the EHR. Following completion of the assessments, the study ID numbers were used to restore the original allocation for analysis. Two independent physicians (1 GP and 1 hospital-based rheumatologist), not involved in the project and without prior experience using the AI system, independently evaluated each note while blinded to implementation status. The hospital-based rheumatologist was selected because of extensive clinical experience with referrals from general practice to both hospital and rehabilitation services, providing substantial familiarity with documentation from primary care despite not working in general practice.

External reviewers rated the final clinical notes after GP review, editing, and approval, corresponding to the version stored in the EHR rather than the initial AI-generated draft. Clinical note quality, structure, and clinical clarity were rated using the same 5-point Likert scales as those used by the participating GPs. Each note was evaluated independently by both reviewers. The extent of GP editing of the AI-generated drafts was not quantified.

### Statistical Analysis

Analyses were conducted at the GP level to avoid inflation of precision due to multiple notes per clinician. For each GP, mean scores were calculated across all available preimplementation notes and all postimplementation notes for each outcome.

The primary analyses used 2-tailed paired *t* tests on GP-level mean differences. Although the underlying variables were measured on ordinal Likert scales, averaging approximately 12 observations per GP resulted in approximately continuous GP-level outcomes. Inspection of the paired differences showed no evidence of marked skewness or influential outliers. Therefore, 2-tailed paired *t* tests were considered appropriate. Wilcoxon signed-rank tests were additionally performed as sensitivity analyses and yielded consistent conclusions.

Given the exploratory nature of the evaluation, *P* values were interpreted descriptively and presented alongside mean differences, 95% CIs, and paired-samples Cohen *d*_z_, calculated as the mean paired change divided by the SD of the paired changes.

For blinded external assessment, mean preimplementation and postimplementation scores were calculated by averaging ratings across both reviewers prior to GP-level aggregation. Interrater reliability between the 2 blinded reviewers was assessed using intraclass correlation coefficients (ICCs; using a 2-way random-effects model with absolute agreement).

### Ethical Considerations

The project was conducted as a pragmatic pre-post quality improvement evaluation within routine clinical practice. According to Danish regulation, the project was classified as a quality improvement initiative and therefore did not require approval from a regional research ethics committee. A data protection impact assessment (DPIA) and a data processing agreement were completed before implementation. All data were handled in accordance with Danish data protection regulations and the GDPR.

Prior to participation, patients received information about the AI-assisted documentation system through information screens in the waiting area, written information available in the consultation room, and verbal information provided by the GP. Patients were given the opportunity to ask questions and decline the use of Noteless before the consultation. Patients verbally consented to participation prior to the consultation. Participation could be declined without any consequences for care. Consultation data were contractually prohibited from being used for AI model training. No identifiable patient information was stored in the study dataset or shared for external assessment.

## Results

### Study Sample and Data Completeness

A total of 20 GPs from 7 general practice clinics participated in the study. Due to incomplete registrations or inconclusive outcome ratings, not all GPs contributed the intended 12 notes in both periods. In total, 475 clinical notes were included in the analysis (preimplementation notes: n=239; postimplementation notes: n=236). One preimplementation note and 2 postimplementation notes were missing because 2 GPs did not reach 12 documented consultations, and 2 postimplementation notes were excluded due to incomplete or inconclusive ratings. The median number of evaluable notes per GP was 12 (IQR 12-12) both before and after the implementation. Analyses were therefore conducted at the GP level using available data.

All participating GPs contributed to the self-assessment analyses. All included notes were available for blinded external assessment by 2 independent physicians.

### Self-Assessed Documentation Quality and Time Use

Descriptive self-assessed documentation scores before and after implementation of AI-assisted documentation are summarized in Table 1.

**Table 1.** Mean self-assessed and blinded documentation scores before and after implementation of AI-assisted documentation^a^.

Outcomes	Self-assessed documentation, mean (SD)		Blinded documentation, mean (SD)	
	Preintervention score	Postintervention score	Preintervention score	Postintervention score
Quality	4.02 (0.44)	4.05 (0.59)	4.29 (0.40)	4.53 (0.20)
Structure	3.99 (0.49)	4.45 (0.57)	4.30 (0.37)	4.60 (0.18)
Clinical clarity	4.10 (0.46)	4.42 (0.57)	4.42 (0.39)	4.73 (0.19)
Documentation time category	2.85 (0.46)	2.29 (0.68)	—^b^	—

a Values are presented as mean (SD) based on general practitioner (GP)–level mean scores from 20 participating GPs. Quality, structure, and clinical clarity were rated on 5-point Likert scales (1=“lowest” to 5=“highest”). Documentation time was recorded using 5 ordinal categories (1=“<1 minute” to 5=“>5 minutes”). Self-assessments were completed by participating GPs; blinded assessments were performed by 2 independent physicians.

b Em dashes indicate that documentation time category was not assessed in the blinded external assessment.

At a descriptive level, mean self-assessed overall documentation quality showed minimal change after implementation, whereas structural quality and clinical clarity increased. The documentation time category decreased, indicating shorter self-reported documentation time following AI-assisted note generation.

GP-level paired analyses are presented in Table 2. Structural quality was higher after implementation, with a mean increase of 0.45 points (95% CI 0.05-0.84; Cohen d_z_=0.50). Clinical clarity also appeared higher after implementation, with a mean increase of 0.31 points (95% CI −0.01 to 0.62; Cohen d_z_=0.45). No statistically significant change was observed for overall self-assessed quality.

Self-assessed documentation time category was lower after implementation at the GP level (mean change −0.55; 95% CI −0.94 to −0.16; Cohen d_z_=−0.61). A mean change of −0.55 categories corresponded approximately to a shift of half a category toward shorter documentation times at the GP level.

Sensitivity analyses using the Wilcoxon signed-rank test yielded results consistent with the primary 2-tailed paired *t* test analyses. Improvements in documentation structure (*P*=.03) and documentation time category (*P*=.02) remained statistically significant, whereas overall documentation quality (*P*=.60) and clinical clarity (*P*=.08) remained nonsignificant, supporting the robustness of the primary analyses.

**Table 2.** General practitioner (GP)–level paired pre-post analyses of self-assessed documentation outcomes^a^.

Outcomes	Mean change (SD)	95% CI	Cohen *d*_z_	*P* value^b^
Quality	+0.02 (0.80)	–0.33 to 0.37	0.03	.91
Structure	+0.45 (0.90)	0.05 to 0.84	0.50	.04
Clinical clarity	+0.31 (0.69)	–0.01 to 0.62	0.45	.06
Documentation time category	–0.55 (0.89)	–0.94 to –0.16	–0.61	.01

a Analyses were conducted at the GP level (N=20) using 2-tailed paired *t* tests, with each GP’s mean score across available consultations. Mean change is reported with 95% CIs. Paired-samples Cohen *d*_z_ is reported as the standardized effect size.

b *P* values are reported for exploratory purposes only.

### Associations Between Documentation Time and Perceived Quality

Exploratory analyses showed a negligible association between documentation time category and self-assessed note quality (GP-level scatter plots; *R*^²^=0.01-0.05), suggesting that longer documentation time was not associated with higher perceived quality.

### Blinded External Assessment of Documentation Quality

Blinded external assessments are summarized in Table 1.

For external reviewer 1, mean scores increased across all domains following implementation: overall quality from 4.00 to 4.63, structural quality from 4.00 to 4.66, and clinical clarity from 4.26 to 4.75.

For external reviewer 2, mean overall quality changed from 4.58 before implementation to 4.44 after implementation, while structural quality changed from 4.60 to 4.52. Clinical clarity increased from 4.58 to 4.70. Thus, reviewer-specific ratings were not fully concordant: reviewer 1 rated all 3 domains higher after implementation, whereas reviewer 2 rated clinical clarity higher but overall quality and structure slightly lower after implementation. Accordingly, the blinded external assessment should be regarded as supportive rather than confirmatory evidence.

When combining ratings from both blinded reviewers (Table 3), mean overall quality increased from 4.29 before implementation to 4.53 after implementation (mean change +0.24). Structural quality increased from 4.30 to 4.60 (+0.30), and clinical clarity increased from 4.42 to 4.73 (+0.31).

**Table 3.** Blinded external assessment of documentation quality and interrater reliability^a^.

Outcomes	Pre-post score change, mean (SD)	95% CI	Cohen *d*_z_	Intraclass correlation coefficient (95% CI)
Quality	0.24 (0.33)	0.08-0.37	0.68	0.19 (0.10-0.28)
Structure	0.30 (0.31)	0.17-0.44	0.98	0.24 (0.15-0.32)
Clinical clarity	0.31 (0.30)	0.18-0.44	1.04	0.34 (0.25-0.42)

a Interrater reliability between 2 blinded reviewers was assessed using intraclass correlation coefficients (2-way random effects, absolute agreement). Higher values indicate greater agreement. Analyses were performed at the general practitioner (GP) level (N=20), using each GP’s mean score across available consultations.

### Interrater Reliability

#### Overview

Interrater reliability between the 2 blinded external reviewers is presented in Table 3. Interrater reliability was low (ICC 0.19-0.34 across domains), indicating substantial between-rater variability in absolute ratings. Despite this variability, the combined reviewer scores were higher after implementation across all assessed domains. However, reviewer-specific ratings were not fully concordant, and the blinded external assessment should therefore be interpreted cautiously.

#### Perceived Impact on Time Available for Patient Care

In the postimplementation audit, GPs reported a mean score of 3.86 (SD 1.08) for perceived increased time available for direct patient care. As no corresponding preimplementation measure was available, this outcome is reported descriptively only.

#### Technology Acceptance and Usability

Technology acceptance was high across all TAM domains. Mean perceived usefulness was 3.76 (SD 0.99), and perceived ease of use was 4.19 (SD 0.78). Attitude toward use was similarly positive, with a mean score of 3.97 (SD 0.97), while behavioral intention to use the system was 3.76 (SD 1.10).

SUS scores indicated good overall usability, with a mean score of 77.5 (SD 14.6), corresponding to high perceived ease of use and learnability.

## Discussion

### Overview

This study evaluated the implementation of an AI-assisted documentation system in routine Danish general practice, combining self-assessment by participating GPs with blinded external evaluation. Overall, the findings suggest that AI-assisted documentation appeared feasible to integrate into everyday clinical work and was associated with high clinician acceptance, lower self-reported documentation time categories, and maintained or modestly improved perceived documentation quality. Blinded external assessment provided limited supportive evidence for these findings but should be interpreted cautiously given the low interrater reliability and reviewer-level differences. These findings should be interpreted within the context of an exploratory pragmatic pre-post evaluation and an evidence base that is still evolving for large language model–based ambient documentation systems.

Although the study was designed as a pragmatic quality improvement evaluation, we included GP-level paired pre-post analyses to provide transparent quantitative support for the observed changes. By defining the GP as the primary analytic unit and summarizing each clinician’s documentation across multiple consultations, we aimed to balance methodological rigor with the exploratory and practice-oriented nature of the study, in line with recommendations for real-world digital health evaluations.

### Principal Findings

Self-assessed documentation quality, structure, and clinical clarity appeared higher following implementation of AI-assisted documentation, while the self-reported documentation time category decreased from 2.85 to 2.29, corresponding to a shift toward shorter documentation time. The largest observed differences were found for structural quality and clinical clarity, whereas overall perceived quality showed minimal change. Importantly, these patterns were also observed by blinded external reviewers, who reported similar directional improvements in overall quality, structure, and clinical clarity.

The overall pattern observed in the blinded assessments was broadly consistent with the GPs’ self-assessments, but reviewer-specific results were not fully concordant. One reviewer rated all domains higher after implementation, whereas the second reviewer rated overall quality and structure slightly lower and clinical clarity higher. Together with the low interrater reliability, this indicates that the external assessment should not be interpreted as confirmatory evidence of improved documentation quality. Rather, it provides limited supportive evidence that implementation was not associated with a clear deterioration in clinical clarity or overall documentation quality, while highlighting the subjective nature of evaluating narrative clinical notes.

### Documentation Time and Quality

The observed reduction in self-reported documentation time categories without a corresponding decline in perceived quality is clinically relevant. Exploratory analyses demonstrated a negligible association between documentation time and perceived documentation quality, indicating that longer documentation time did not translate into higher-quality notes. This challenges the implicit assumption that perceived documentation quality necessarily depends on time investment and suggests that AI-assisted drafting may support more efficient documentation workflows without sacrificing clinical clarity.

These findings align with emerging international evidence showing that AI-assisted documentation systems, including ambient AI and AI-powered voice-to-text solutions, have been associated with reductions in perceived administrative burden while maintaining or improving documentation quality and clinician workflow [1,4-7,11]. Prior studies have reported reduced perceived workload, improved efficiency, and stable documentation quality in primary care and outpatient settings, although most have relied primarily on self-reported outcomes. By incorporating blinded external evaluation, the present study extends the literature with a more robust assessment of documentation quality under routine clinical conditions.

Our findings are also consistent with a recent longitudinal mixed methods implementation study from Dutch general practice, which similarly reported reduced perceived documentation burden and improved workflow following implementation of an ambient AI scribe. However, the authors also identified unintended consequences, including occasional inaccuracies in AI-generated summaries, challenges during sensitive consultations, and potential interference with clinicians’ clinical reasoning [12]. Likewise, the recent *EBioMedicine* systematic review concluded that AI-assisted documentation systems show considerable potential to improve efficiency and patient-centered care while emphasizing persistent concerns regarding transcription errors, safety, and the limited generalizability of the current evidence base [6]. This distinction is also reflected in the recent *The Lancet Primary Care* review, which concluded that clinician satisfaction and perceived reductions in documentation burden have been reported more consistently than objectively measured improvements in efficiency. The present findings are consistent with this pattern, as documentation time was assessed using self-reported documentation time categories rather than objective time measurements [13]. Taken together, these findings suggest that AI-assisted documentation is promising, but that continued evaluation in routine clinical practice remains essential as these technologies evolve.

### Acceptance, Usability, and Implementation Considerations

TAM and SUS results indicated high acceptance and perceived usability of the AI-assisted documentation system. High scores for perceived usefulness and ease of use are consistent with established determinants of digital health adoption and suggest that clinicians experienced the system as supportive rather than disruptive to clinical practice [9,10]. Behavioral intention to use also remained high, indicating potential for sustained use beyond the study period.

Implementation of AI-assisted documentation in general practice raises important considerations related to trust, transparency, and data governance. Previous qualitative research has shown that clinicians’ willingness to adopt generative AI tools depends on perceived control, reliability, and clarity regarding data handling [9]. Similarly, a recent qualitative study of AI scribes in Norwegian general practice found that clinicians generally perceived ambient documentation systems as supportive of clinical workflows, while also highlighting concerns regarding accuracy, sensitive consultations, and maintaining clinician oversight. These findings are consistent with the implementation considerations identified in the present study [14]. Similarly, patient perspectives emphasize the importance of transparency and appropriate data governance in the use of AI-based solutions in primary care [15]. The explicit deletion of audio data and short retention period of generated notes in the present implementation may therefore have contributed to both clinician and patient acceptance.

### Strengths and Limitations

A key strength of this study is the combination of a pre-post design with blinded external assessment, reducing reliance on self-reported outcomes alone. The use of validated instruments (TAM and SUS) further supports the reliability and interpretability of the findings. Additionally, the study was conducted under routine clinical conditions within a nontechnology-selected group of GPs, enhancing ecological validity. However, formal measures of practice characteristics, including digital maturity, were not collected, limiting the assessment of transferability to other primary care settings.

Several limitations should be considered. The exploratory pre-post design limits causal inference and observed changes may partly reflect learning effects or increased familiarity with documentation practices over time. Because reasons for excluding consultations were not recorded, we were unable to assess whether the case mix differed between the preimplementation and postimplementation periods. The 2-week familiarization period may have been insufficient for all clinicians to fully integrate the system into their workflows, and longer exposure could yield different assessments of usability and efficiency. Documentation time was assessed using self-reported ordinal time categories rather than objectively recorded time measurements. The extent of GP editing of AI-generated drafts was not quantified, and we did not assess lexical characteristics such as note length, number of unique words, or documentation complexity. These measures may provide important complementary information in future evaluations of AI-assisted documentation systems. Consequently, the observed reduction should be interpreted as reflecting clinicians’ perceived documentation burden rather than precise time savings in minutes. This distinction is particularly important given that a recent comprehensive review suggests that perceived improvements in documentation burden have been reported more consistently than objectively measured gains in efficiency for AI-assisted documentation systems [13]. Interrater reliability between the 2 blinded external reviewers was low, indicating substantial variability in absolute ratings. In addition, reviewer-specific ratings were not fully concordant, as 1 reviewer rated all domains higher after implementation, whereas the other rated overall quality and structure slightly lower and clinical clarity higher. The blinded external assessment should therefore be interpreted as limited supportive evidence rather than confirmatory evidence of improved documentation quality. We did not formally assess the success of reviewer blinding and therefore cannot exclude the possibility of partial unblinding.

This variability may partly reflect the inherently subjective nature of assessing narrative clinical documentation, even among experienced clinicians. In addition, the 2 reviewers represented different clinical contexts, with 1 working in hospital-based care and the other in general practice. Conceptions of high-quality documentation may therefore differ due to distinct workflows and informational needs rather than differences in documentation quality per se. Hospital-based documentation often emphasizes completeness and detailed clinical information, whereas general practice documentation typically prioritizes concise, structured notes that support rapid overview, continuity of care, and efficient clinical decision-making. Finally, although AI-assisted documentation systems show considerable promise, robust longitudinal real-world evidence for newer large language model–based systems remains limited. Future studies should therefore evaluate not only documentation quality and efficiency but also potential unintended consequences, including effects on clinical reasoning, patient communication, equity, and documentation accuracy as these technologies continue to evolve [13].

### Implications and Future Research

Beginning January 1, 2027, Danish patients are expected to have online access to general practice clinical notes in a manner comparable to current access to hospital records. In this context, the quality, structure, and clinical clarity of documentation become increasingly important not only for interprofessional communication but also for patient understanding. The consistently high scores for structure and clinical clarity observed in this study suggest that AI-assisted documentation may support the production of more transparent and patient-readable clinical notes, extending potential benefits beyond efficiency gains alone.

Future research should include controlled study designs, longer follow-up periods, and assessment of patient perspectives, consultation-level characteristics, and clinical outcomes. Future studies should also incorporate objectively measured documentation time alongside clinician-reported outcomes to better distinguish perceived from actual workflow improvements. Further work is needed to evaluate how AI-assisted documentation influences continuity of care, medico-legal documentation quality, interprofessional communication, documentation accuracy, clinical reasoning, and equity across patient populations.

### Conclusions

Implementation of AI-assisted clinical documentation in Danish general practice was associated with lower self-reported documentation time categories and no clear deterioration in documentation quality. Clinician acceptance and perceived usability were high. Blinded external assessment provided limited supportive evidence, but low interrater reliability and reviewer-level divergence warrant cautious interpretation. Larger controlled studies with objective time measurement, patient-level and consultation-level data, and longer follow-up are needed to evaluate effectiveness, safety, documentation accuracy, and potential unintended consequences in routine primary care.

## Abbreviations

DPIA: data protection impact assessment
EHR: electronic health record
EU: European Union
GDPR: General Data Protection Regulation
GP: general practitioner
ICC: intraclass correlation coefficient
SUS: System Usability Scale
TAM: technology acceptance model

## References

[1] Olson KD, Meeker D, Troup M (2025). Use of ambient AI scribes to reduce administrative burden and professional burnout. JAMA Netw Open.

[2] Bundy H, Gerhart J, Baek S (2024). Can the administrative loads of physicians be alleviated by AI-facilitated clinical documentation?. J Gen Intern Med.

[3] Rotenstein LS, Apathy N, Holmgren AJ, Bates DW (2023). Physician note composition patterns and time on the EHR across specialty types: a national, cross-sectional study. J Gen Intern Med.

[4] Bracken A, Reilly C, Feeley A, Sheehan E, Merghani K, Feeley I (2025). Artificial intelligence (AI) - powered documentation systems in healthcare: a systematic review. J Med Syst.

[5] Guo Y, Wang J, Hu D (2026). Evaluating ambient artificial intelligence documentation: effects on work efficiency, documentation burden, and patient-centered care. J Am Med Inform Assoc.

[6] Alboksmaty A, Aldakhil R, Hayhoe BW, Ashrafian H, Darzi A, Neves AL (2025). The impact of using AI-powered voice-to-text technology for clinical documentation on quality of care in primary care and outpatient settings: a systematic review. EBioMedicine.

[7] Shah SJ, Devon-Sand A, Ma SP (2025). Ambient artificial intelligence scribes: physician burnout and perspectives on usability and documentation burden. J Am Med Inform Assoc.

[8] Falcetta FS, de Almeida FK, Lemos JC, Goldim JR, da Costa CA (2023). Automatic documentation of professional health interactions: a systematic review. Artif Intell Med.

[9] Blease C, Garcia Sanchez C, Locher C, McMillan B, Gaab J, Torous J (2025). Generative artificial intelligence in primary care: qualitative study of UK general practitioners’ views. J Med Internet Res.

[10] Zhao J, Liu H, Chen Y, Song F (2025). Application of artificial intelligence tools and clinical documentation burden: a systematic review and meta-analysis. BMC Med Inform Decis Mak.

[11] Kuhn T, Basch P, Barr M, Yackel T, Medical Informatics Committee of the American College of Physicians (2015). Clinical documentation in the 21st century: executive summary of a policy position paper from the American College of Physicians. Ann Intern Med.

[12] van Linschoten RC, van Loon CM, Joanknecht L, Bischoff EW (2026). Ambient scribe in general practice: a multi-perspective before-after longitudinal mixed-methods study. NPJ Digit Med.

[13] Laranjo L, Tudor Car L, Payne RE, Neves AL, Kidd M, Jaime Miranda J (2026). Artificial intelligence in primary care: innovation at a crossroads. Lancet Prim Care.

[14] Nassehi D, Hetlevik Ø, Breivold J (2026). Exploring AI scribes in Norwegian general practice: a qualitative individual interview study. Scand J Prim Health Care.

[15] Mikkelsen JG, Sørensen NL, Merrild CH, Jensen MB, Thomsen JL (2023). Patient perspectives on data sharing regarding implementing and using artificial intelligence in general practice - a qualitative study. BMC Health Serv Res.

